# Exploring the ’EarSwitch’ concept: a novel ear based control method for assistive technology

**DOI:** 10.1186/s12984-024-01500-z

**Published:** 2024-12-02

**Authors:** Anna C. Hoyle, Richard Stevenson, Martin Leonhardt, Thomas Gillett, Uriel Martinez-Hernandez, Nick Gompertz, Christopher Clarke, Dario Cazzola, Benjamin W. Metcalfe

**Affiliations:** 1 Department of Electronic and Electrical Engineering, University of Bath, Bath, UK; 2 Department for Health, University of Bath, Bath, UK; 3 EarSwitch Ltd., Manchester, UK; 4 Department of Computer Science, University of Bath, Bath, UK; 5 Centre for Analysis of Motion and Entertainment Research and Application (CAMERA), University of Bath, Bath, UK; 6 Bath Institute for the Augmented Human (IAH), University of Bath, Bath, UK; 7 School of Engineering and Physical Science, Heriot-Watt University, Edinburgh, UK

**Keywords:** Assistive technology, Novel input, Tensor Tympani, Motor neurone disease, ALS, Earables, Ear rumble, EarSwitch

## Abstract

**Background:**

Loss of communication with loved ones and carers is one of the most isolating and debilitating effects of many neurological disorders. Assistive technology (AT) supports individuals with communication, but the acceptability of AT solutions is highly variable. In this paper a novel ear based control method of AT, the concept of ’EarSwitch’, is presented. This new approach is based on detecting ear rumbling, which is the voluntary contraction of the tensor tympani muscle (TTM), resulting in observable movement of the eardrum and a dull rumbling sound. ’EarSwitch’ has the potential to be a discreet method that can complement existing AT control methods. However, only a subset of the population can ear rumble and little is known about the ability of rumbling in populations with neurological disorders.

**Methods:**

To explore the viability of the ’EarSwitch’ concept as an AT control method we conducted in-depth online surveys with (N=1853) respondents from the general population and (N=170) respondents with self-declared neurological disorders including Motor Neurone Disease (MND) and Multiple Sclerosis (MS).This is the largest ever study to explore ear rumbling and the first to explore whether rumbling is preserved among individuals with neurological disorders. In addition, we validated rumbling, and investigated usability of the ’EarSwitch’ concept as a control input, using in-person otoscopic examination with a subset of participants.

**Results:**

A significant proportion of the population with neurological disorders could benefit from ’EarSwitch’ controllable AT. The upper bound prevalence of the ability to rumble without accompanying movements was 55% in the general population, 38% in the neurological population, and 20% of participants with MND (N=95) reported this ability. During the validation procedure, participants achieved high accuracy in self-reporting the ability to rumble (80%) and proved concept of using the ’EarSwitch’ method to control a basic interface.

**Discussion:**

’EarSwitch’ is a potential new AT control method control, either by itself or as a supplement to other existing methods. Results demonstrate self-reported ear rumbling is present among patients with different neurological disorders, including MND. Further research should explore how well the ability to rumble is preserved in different types and stages of neurological disorders.

**Supplementary Information:**

The online version contains supplementary material available at 10.1186/s12984-024-01500-z.

## Background

Neurological disorders refer to a wide range of conditions that affect the nervous system, impacting different aspects of a person’s functioning. For example, one of the most common neurological disorders affecting mobility is Multiple Sclerosis (MS), in which the immune system mistakenly attacks the myelin sheets covering the nerve fibers. This disrupts the transmission of nerve signals and causes symptoms such as muscle weakness and difficulty with coordination and balance. Another example, are motor neurone diseases (MNDs) which affect mobility as a result of damage to the motor neurons responsible for controlling voluntary muscle movement. Irrespective of the underlying pathology, there are many other neurological disorders that cause impaired mobility or speech which leads to a significant decline in quality of life [1] threatening mental health for both patients [2] and caretakers [3]. Unfortunately, there are no known cures for these neurological disorders [4] and it is common that individuals lose their independence as the disorders progress, becoming entirely dependent upon their caregivers. One solution that can prolong an individual’s independence, is the use of assistive technology.

Assistive technology (AT) includes wheelchairs [5, 6] and robotics arms [7, 8] to help with mobility and feeding which allows an individual to regain autonomy and Augmentative and Alternative Communication (AAC) systems [9, 10] to supplement an individual’s ability to communicate and stay connected with both people and technology. Both of these help to increase an individual’s inclusion within society and their chances of employability to be able to support themselves. Several assistive devices are available, such as alternative mice [11, 12] and keyboards [13], to help individuals control computers and interact with digital interfaces including smart home devices such as televisions and lights which can be controlled through environmental control systems that provide individuals with greater control over their immediate environment [14].

There is a broad landscape of AT devices available to accommodate the wide range of user needs because the level of preserved muscle function varies widely among diseases and among individuals (see Table 1). Furthermore, the choice of input for AT depends on the specific function of the device and the circumstances in which the device is planned to be used. Factors such as speed, portability, comfort, aesthetics, effectiveness, calibration time, fatigue when using the device, cost, and usability independently influence the selection of the control input for an assistive device [15–17]. The broad landscape of AT devices helps to ensure at least one device is available for any given individual, and devices can be used in a complementary multimodal manner to maximise the communication bandwidth and extend individual input preferences through input redundancy and variability [18]. However, individuals will abandon devices that do not meet their needs or which have poor device performance [19, 20], which for AT devices can be user dependent. As a result, it is important to explore and develop alternative AT input devices that can complement existing devices and help to fill gaps in the AT landscape.

**Table 1:** Comparison of control methods of AT for patients with neurological disorders affecting either mobility or communication. EOG=Electrooculogram, EEG=Electroencephalogram, EMG=Electromyography

Control type	Working principle	Strength	Limitations
Eye-gaze	Uses a person’s eye movements for control sensed by either a camera [5, 9] or EOG [21–23]	Usable with severe conditions [8, 24]	Fatigue [25], needs visual attention
Brain signals	Based on decoding electrical signals produced by the brain, sensed by electrodes placed on the head. Either invasively under the skull [26] or non-invasively on the scalp using EEGs [10, 27].	More natural control [28], usable with severe conditions [27, 29]	If non-invasive low accuracy on complex tasks, expensive [30], pre-training needed [31], mentally exhausting [32]
Hand/facial muscles sensed by EMG	Using muscles in hand [33] and face [34, 35] that still at least partially work sensed by EMG	Easy to use, controlled by simple movements, such as eyebrow raise [36]	Not suitable in severe conditions, difficulty of correct electrode placement [35]
Head/chin movement	Either following movement of target fixed on forehead [37] or placing chin on sensors and moving around (joystick) [38–40]	Low cost, high accuracy for controlling direction	Fatigue, can not be used with severe conditions, or with conditions with tremors
Tongue movement	Using tongue movements directed towards sensors placed on the top of the mouth [41, 42]	Usable with severe conditions [43], invisible control, range of inputs [43]	Uncomfortable
Sip and puff switch	Controlling by breath sensed by pressure sensors [6, 44, 45]	Easy to use, portable	Not suitable if respiratory or pulmonary functions are affected, needs frequent maintenance (cleaning), obstructive
Tooth-click controller	Using tooth-clicking for control sensed by either an accelometer placed behind the ear detecting jaw vibrations [46] or detecting the sound of the clicking [47]	Easy to use, comfortable, easy to learn	Not suitable in severe conditions
Voice control	Using speaking or sounds for control [12, 48, 49]	High speed [49]	Not suitable if speech is impaired [50], not practical in social settings as other sounds can interfere, and could disturb others

### Tensor tympani muscle

**Fig. 1:**
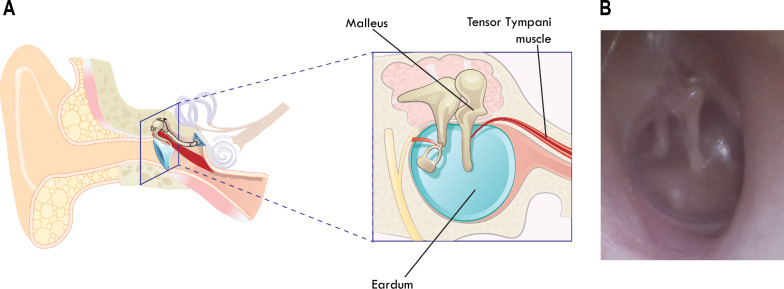
**A** Anatomical structure of the middle ear with the middle ear muscles. The tensor tympani is attached to the malleus and by contracting the muscle, it pulls the eardrum backwards. **B** Photo of the eardrum, taken by an otososcope held in the ear canal

An input mechanism that has yet to be explored in the context of AT is voluntary control of the tensor tympani muscle (TTM). The TTM is a small muscle located in the middle ear (Fig. 1) and is innervated by a branch of the mandibular division of the trigeminal nerve (5th cranial nerve) [51]. When contracted the muscle applies force to the manubrium of the malleus, pulling the eardrum inward [52] which some experience as a rumbling sensation in the ear, commonly referred to as ear rumbling. While the exact role of the TTM is still not fully understood [53], prior patent application [54] and following research [55] has shown how voluntary ear rumbling can be used as a human-computer interaction input technique.

From an AT perspective, rumbling presents a unique opportunity. Middle ear muscles active during rapid eye movement (REM) sleep are preserved late in ALS [56], and the controlling nerve may emerge above the area of damage in brainstem strokes. Consequently, this implies that TTM could serve as a potential alternative to existing AT control methods that lose utility as conditions progress to the later stages. In addition, the act of rumbling is an invisible action to external observers which is subtle and discreet, therefore not drawing attention. From a technical perspective, the visible movement of the eardrum creates different opportunities for sensing, such as a camera placed in the ear canal [54] or sensing pressure change in the ear canal [55] which can be integrated into commonly worn, and socially acceptable ear-based form factors [57]. Ear rumbling may be used as a binary switch (either rumbling or not), and thus be used in a similar fashion to existing assistive technology switches. One example of a binary switch controlled assistive technology is the on-screen scanning keyboard [45, 58]. The keyboard appears on the screen, typically as a grid of keys, a scanning process then highlights or selects keys or groups of keys in a systematic manner, usually row by row or column by column. The user then activates a switch or button to select the highlighted key or group of keys. This switch can be a physical button, a sip-and-puff device, or any other accessible input method. As the ear is not commonly used for AT devices, there is a high potential that it can be used in a complementary nature with existing AT techniques (e.g., eye tracking).

The knowledge that some people can voluntarily contract their TTM (ear rumble) has been long known [59], however there is little information about the percentage of the population with the ability to rumble or how well the control of the TTM is preserved, if at all, with neurological disorders. Röddiger et al. investigated prevalence using an online survey of 192 participants and found that 43.2% self-reported the ability to rumble [55]. From the participants completing the survey, 16 people who self-reported the ability to rumble were invited to the lab, validation was performed with an otoscope looking at the eardrum, and visible movement was observed in all 16 participants. This suggest individuals are self-aware of their own ability to rumble. However, to date no study has investigated whether the ability to rumble is maintained in patients with neurological disorders. The aim of this study is to explore the possibility of using rumbling as an input for AT, a concept commercially explored by EarSwitch Ltd. We complete the first study exploring whether ear rumbling is preserved among patients with neurological disorders, such as MND and how well people can self-report their ability to rumble. Participants with neurological disorders were also asked about their use of assistive devices, including the type and purpose of usage. Although this aspect was not the primary focus of the study, the amassed data could be among the most extensive datasets available on this subject.

## Methods

### Study design

The study was divided into 3 phases. Phase 1 involved an anonymous online survey of healthy adults recruited through social media and word of mouth. Phase 2 was an extended version of the Phase 1 survey conducted with adults who self-declared having some sort of neurological condition affecting motor function. These participants were primarily recruited through relevant associations, such as Motor Neurone Disease Association (MNDA, UK) and Centre for Augmentative and Alternative Communication (Centre for AAC communication, UK). The recruitment materials did not provide information about the study’s purpose. However, links to recruitment were also shared in online articles discussing the project, on the social media channels of the University of Bath and EarSwitch Ltd (a company developing AAC using ear rumbling who were co-Investigators on this project) and a link was subsequently shared on a popular sub-Reddit dedicated to ear rumblers. Individuals under 18 years of age were excluded from all phases of the study. In Phase 2, participants without a neurological condition affecting motor function were also excluded. In Phase 3, participants who had indicated in the Phase 1 and 2 surveys their willingness to take part in further research, self-reported that they could rumble from both general and neurological groups, and indicated they live in the UK were invited to the University of Bath’s Department for Health research laboratories. They were asked to follow a set protocol and replicate ear rumbling for validation purposes. The movement of the eardrum was visually confirmed using an otoscope placed in the ear canal. Individuals with an obstructed view of their eardrum were excluded from this phase. In ten randomly selected cases, an inter-rater reliability test was run on the assessment of the eardrum movement detection.

The study was approved by the Ethics Committee of University of Bath’s Health Department (Reference number: EP 19/20 077). The surveys for Phases 1 and 2 were conducted online using the accessible platform Online Surveys (Jisc) and were open between 18/10/2020 and 14/12/2021. In Phase 3, the local COVID-19 regulations in place at the time were maintained when entering lab spaces. Participants did not receive reimbursement for participating in any part of the study.

**Fig. 2:**
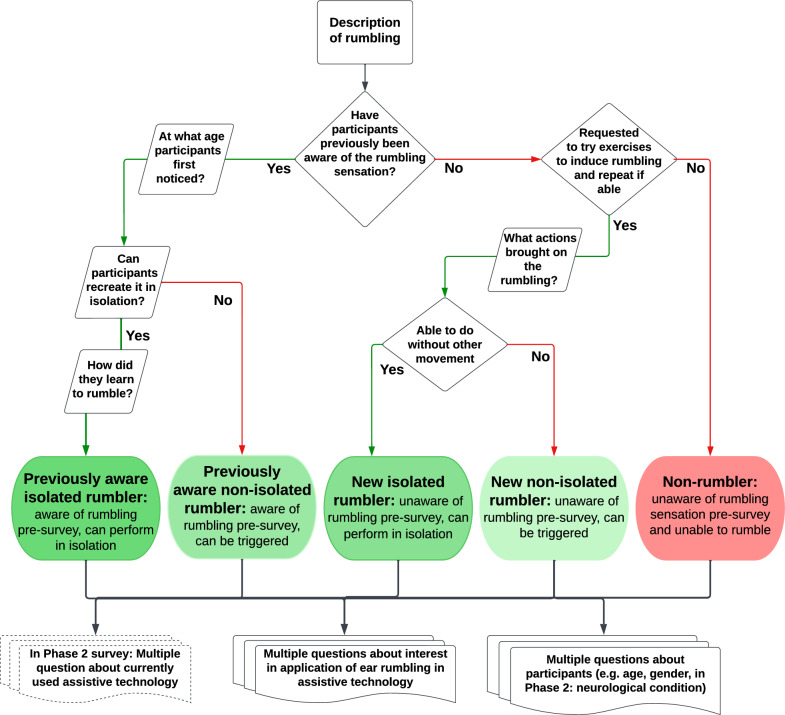
Structure of Phase 1 and Phase 2 surveys. The structure was the same, except that in the Phase 2 survey (the survey among the population with neurological disorders) participants were asked questions regarding their neurological disorder and AT they use

### Procedure

The surveys for phases 1 and 2 consisted of a mixture of multiple choice and open ended questions. These questions aimed to determine whether participants experienced the sensation of ear rumbling and gather specific information about their ability to recreate this feeling. The surveys took approximately 5–10 min to complete, and while most questions were mandatory, participants could skip some questions that did not specifically focus on their ability to rumble, such as their gender and neurological condition. As a preliminary step, a pilot survey was conducted, and after reviewing the responses and feedback, we determined that no changes were necessary for the main survey, so the pilot data was included in the final analysis. The structure of the surveys is shown in Fig. 2 and all survey questions can be found in Additional files 1 and 2. To make sure participants knew what sensation the questions are about, both surveys began with an explanation of ear rumbling:

*‘We know that some people can make a special sound or sensation in their ears either intentionally or when they perform certain movements, such as yawning, closing their eyes tightly, clenching their teeth, or opening their mouth wide. Please note, this is not the same sensation as “equalising” the pressure in your ears (when descending on an aeroplane for example). Nor is it hearing a brief “click” (called a Eustachian tube click). It’s a “rumbling” or “fluttering” noise sensation, like the sound of distant thunder, or a muffling dampening of your hearing. Whether or not you’ve ever experienced this sensation, we’d like to ask you some questions about it. Throughout this survey, we will refer to this sensation as “rumbling”.‘*

Based on their responses to the questions, survey participants were then categorised into five distinct categories based on their ability to ear rumble and whether they were aware of this before or after completing the survey (Fig. 2). Participants were also asked about their age range, gender, interest in controlling devices using rumbling, and willingness to participate in further research (Phase 3). In the Phase 2 survey, participants were asked to describe their neurological condition, if they were assistive device users, and what types they were using and for what function.

#### Validation

**Fig. 3:**
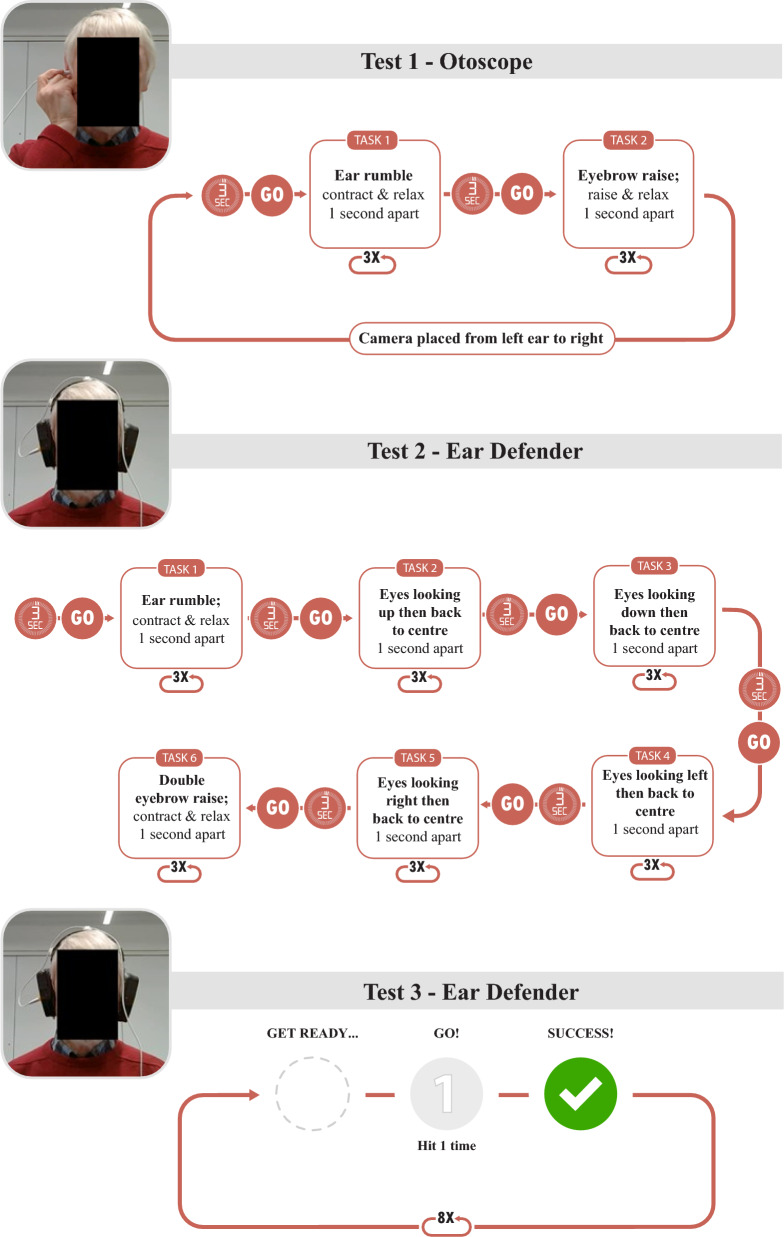
Flowchart of the protocols used for tests in the Validation study. During Test 1 the otoscope was manually held in the ear canal by either the researcher or the participant. In Tests 2 and 3 the otoscope was mounted in a set of modified ear defenders referred to as *Ear Defender* setup. During the tests participants followed instructions from the laptop located in front of them. In the first two tests, the different movements were performed after a three second audible and visual countdown followed by a visual ‘Go!’ and audible ‘bleep’. During Test 3 participants were instructed to react by rumbling upon the appearance of the ’Go! Hit 1 time!’ prompt

Validation was divided into three tests (Fig. 3), each with a distinct standardised protocol. The first two tests focused on the prevalence of isolated rumbling. In both Test 1 and Test 2, the outcome measure was the “presence or absence of eardrum movement”-during isolated rumbling in Test 1, and during isolated rumbling, eye movements, and eyebrow raises in Test 2. The third test focused on the usability of the EarSwitch concept as a binary switch by measuring response time to an onscreen prompt in a pilot study. During each test, individuals sat at a generic workstation facing a laptop computer with a USB connected digital otoscope (Depstech, China) inserted into their ear. In the first two tests, the otoscope was placed in both ears to see if there was movement, and in the third test, the ear with a clearer view was chosen.

In the first two tests, participants were asked to re-create the ear rumbling sensation as well as to perform various eye movements (e.g., raising their eyebrows, looking left and right). These tests were carried out using a custom program created specifically for this project, which displayed graphical instructions on the laptop screen in front of the participant (Fig. 3). In Test 1, to validate the results in an easy and fast way the otoscope was held in the ear canal pointing at the eardrum by either the participant or the researcher. An otoscope mounted in a set of modified ear defenders was used to reduce the camera movement caused from manual holding in Tests 2 and 3 (Fig. 3). In both tests video data gained by the otoscope was recorded and at the same time movement of the eardrum was noted by the researcher from the live video feed as either ‘yes’, ‘no’ or ‘uncertain’. The pilot study in Test 3 was conducted with a subset of participants (n=10), including the first 9 individuals from the general population and the only participant from the neurological group who exhibited visible eardrum movement in Tests 1 and 2. This test was conducted using an internet software (https://www.scanningwizard.com/ [60]). Participants responded to a series of eight visual cues on a laptop by rumbling as soon as possible. Results of the mean reaction time and the number of extra hits (false positive triggers) were displayed on the screen. Participants were then asked to rate on a 1–5 scale how easy they found the task; very easy (1) and very difficult (5).

#### Movement detection

In the first two tests in addition to the movement detection by the researchers during the experiment, three independent raters were asked to evaluate ten randomly selected recorded videos and rate the amount of eardrum movement that could be seen for the first two tests. The raters, whom were researchers unrelated to the project and had no prior experience with otoscope images, were given a brief overview of the project and they were shown examples of participant videos with no movement, one with very small movement, and one with large movement. Each video was shown at least three times, and raters could request it to be shown as many times as needed. The raters evaluated each video with a score of 0–3; no movement at all (score 0), a small amount (score 1), a moderate amount of movement (score 2), and a large amount of movement (score 3). In the third test, a contour-based motion detection algorithm was employed to detect movement; however, the motion detection threshold was not personalised.

#### Data analysis

Statistical analysis of the quantitative results was carried out using IBM SPSS version 26 (IBM, New York, USA). Descriptive statistics (totals and percentages) were calculated to describe and highlight any differences between general and neurological populations and specific ear rumbling groups. A binomial test was used to compare any differences between the researchers assessment and the independent raters subjective assessments of eardrum movement. To determine the reliability of the scoring of eardrum movement during the movement analysis between assessors, an intraclass correlation coefficient analysis was conducted. Statistical significance was set at p <.05. To determine the significance of the difference between the proportion of isolated rumblers and all rumblers among the neurological and general populations, the chi-square test of homogeneity [61] was used. The statistical significance was set at p <.05.

## Results

### Participants

**Fig. 4:**
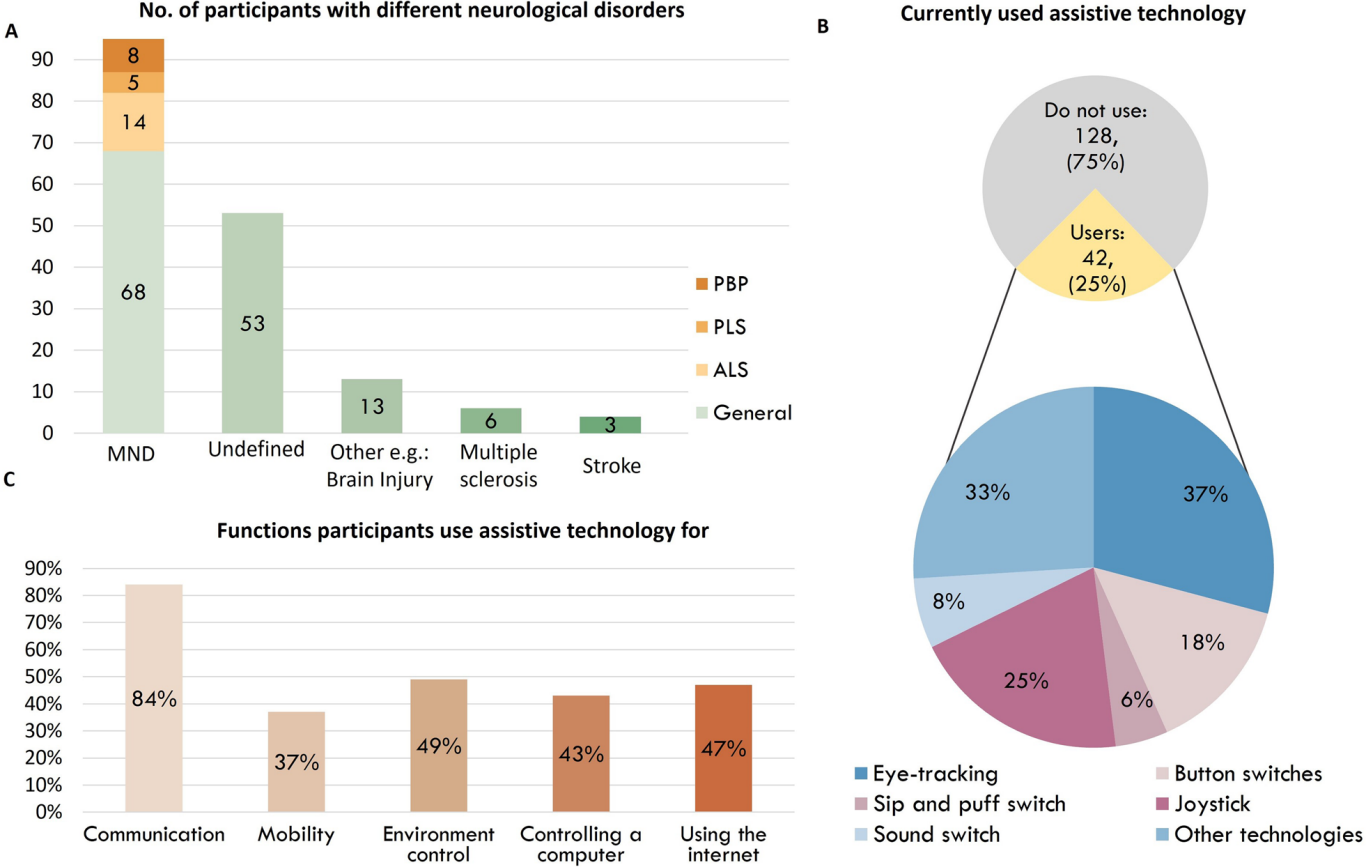
Summary of demographics of participants of the Phase 2 survey with different neurological conditions and current AT users. **A** Age distribution of participants. **B** Distribution of different self-defined neurological disorders. PBP=Progressive bulbar palsy, PLS=Primary Lateral Sclerosis, ALS=Amyotrophic Lateral Sclerosis **C** Types of AT used by participants. **D** Functions participants use AT for

For Phase 1, 1872 individuals agreed to take part in the general population survey. 19 responses were removed for reporting being under the age of 18. A total of 1853 participants from the general population gave their digital consent and completed the online survey. For Phase 2, participants were recruited through connections with 21 AT and MND organizations. 221 individuals agreed to take part, five responses were removed for reporting being under the age of 18. Additionally, 46 participants were excluded from the analysis because the neurological condition they reported did not meet the criteria for a motor neurological condition. A total of 170 participants with a neurological condition gave their digital consent and completed the online survey. 42 participants from the neurological population were already AT users (see Fig. 4 for summary of participant demographics). Due to the COVID-19 pandemic, recruitment for Phase 3, as it took place in person, was particularly challenging, especially among individuals with neurological disorders. For Phase 3, 92 survey participants (88 from the general population, four with neurological conditions) volunteered for the validation study. 22 individuals (20 from the general respondents and two with neurological conditions) were unable to complete the full validation protocol due to an obstructed view (mainly caused by earwax, but in some cases ear hair contributed as well) of both eardrums making the analysis impossible. Test 3 of the validation, designed to demonstrate the proof of concept for using EarSwitch as a binary input, was conducted with 9 individuals from the general population and 1 participant from the neurological group who had Multiple Sclerosis. (The full demographic information of the participants for both surveys and the validation can be found in Appendices.)

### Results of surveys

Results from the surveys in Phases 1 and 2 are presented in Table 2. Among the general population, 85% self-reported the ability to rumble, with 65% able to do so in isolation. In the neurological population, 70% reported the ability to rumble, and 55% could rumble in isolation. This indicates that the proportion of isolated rumblers among all rumblers is lower in the neurological group (55%) compared to the general group (65%). According to the test of two proportions (chi-square test of homogenity) this 10% difference is statistically significant ($p =.029$).

After completing the survey, 28% (20 out of 71) of participants from the neurological population who were not aware of their rumbling ability prior to the survey discovered they could rumble. Among the general population, 48% (259 out of 537) of those who were not previously aware of rumbling newly discovered their ability to rumble.

Data from Table 2 also indicates that participants who discovered their ability to rumble during the surveys were less likely to rumble in isolation. The isolation rate among the newly aware general population was 40% (103 out of 259), compared to 25% (5 out of 20) in the neurological population. These rates are significantly lower than the isolation rates among participants who were already aware of their rumbling ability: 69% (914 out of 1316) in the general population and 60% (60 out of 99) in the neurological population.

Most participants in Phase 2 had some type of MND (N=95) and 20% (19 out of 95) could rumble in isolation. The results of the Phase 2 survey, taking into account the different types of neurological disorders, are shown in Table 3. The full survey results for both Phase 1 and Phase 2 are available in Additional files 3 and 4.

**Table 2:** Results of surveys among the general population (Phase 1) and the population with neurological disorders (Phase 2)

	General population n (%)	Neurological population n (%)
Completed surveys	1853 (100%)	170 (100%)
**Aware of rumbling pre-survey**	**1316 (71%)**	**99 (58%)**
Previously aware isolated rumbler	914 (49%)	60 (35%)
Previously aware non-isolated rumbler	402 (22%)	39 (23%)
**Not aware of rumbling pre-survey**	**537 (29%)**	**71 (42%)**
**Newly aware of rumbling post-survey**	**259 (14%)**	**20 (12%)**
New isolated rumbler	103 (6%)	5 (3%)
New non-isolated rumbler	156 (8%)	15 (9%)
**Total able to rumble in isolation**	**1017 (55%)**	**65 (38%)**
Total able to rumble	1575 (85%)	119 (70%)
**Total non-rumbler**	**278 (15%)**	**51 (30%)**
No. isolated rumblers/No. all rumblers (%)		
**Proportion of those rumblers that can rumble in isolation**	**1017/1575 (65%)**	**65/119 (55%)**

**Table 3:** Results of the survey among the neurological population regarding different disorders

	MND In general	*ALS*	*Bulbar ALS*	*PLS*	*PBP*	MS	Stroke	Other	Did not define
No. partcipants	68	12	2	5	8	6	3	13	53
Able to rumble	36	6	2	2	2	4	3	13	51
Able to rumble in isolation	13	6	0	0	0	2	1	6	37

#### Trigger movements to achieve rumbling and learning to rumble

**Fig. 5:**
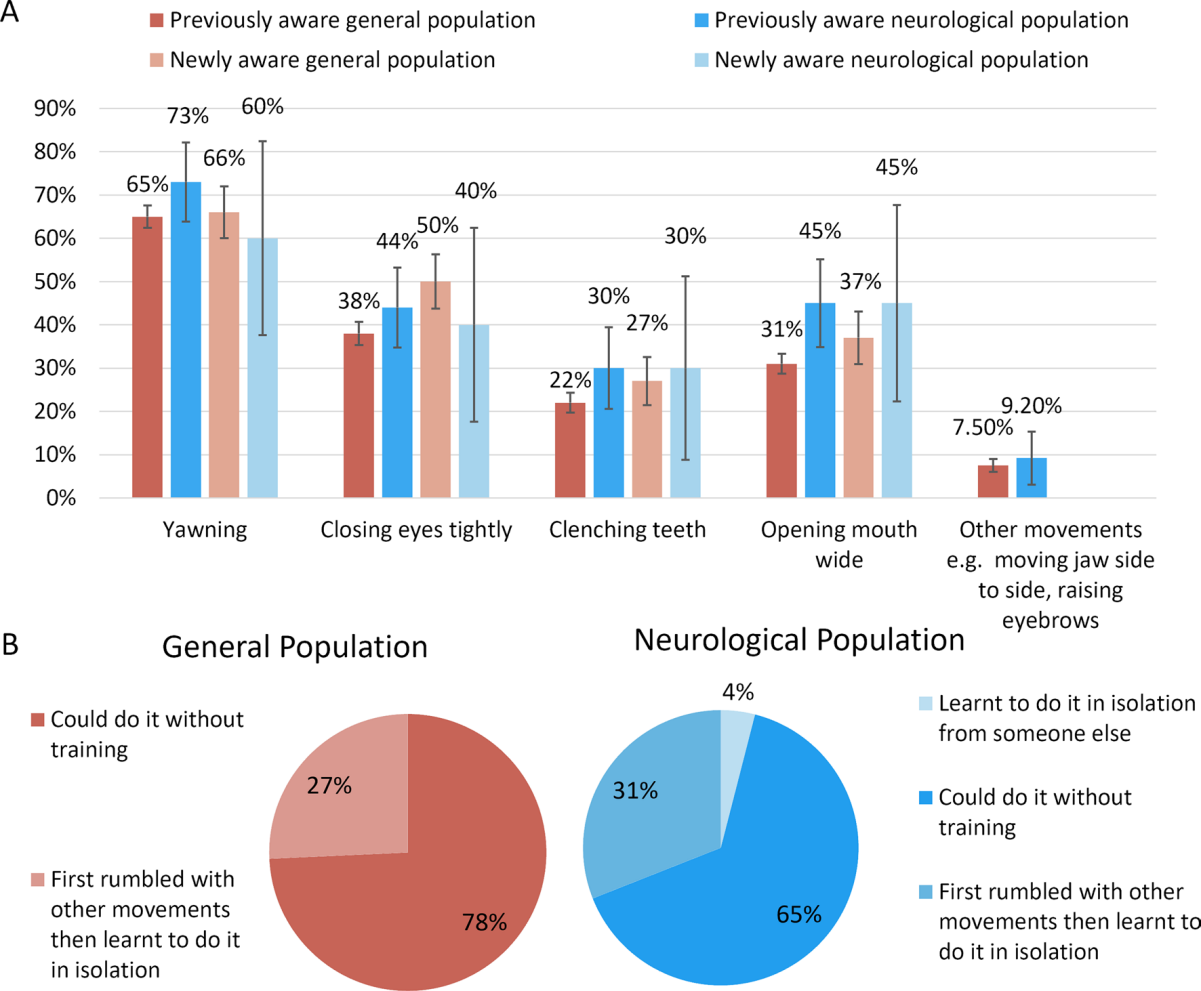
**A** Survey responses indicating the actions that trigger the ear rumbling sensation. The bars represent the percentage of respondents in each group who reported being able to trigger ear rumbling with specific movements. The four respondent groups are: those previously aware of their ability to rumble (before survey) in both the general and neurological populations, and those newly aware after the survey, also in both populations. Error bars show the 95% confidence intervals (calculated using the Clopper-Pearson exact method). **B** Survey responses from participants who were aware of their ability to rumble in isolation, detailing how they acquired this skill

Participants stated that they could bring about the rumbling sensation whilst performing other movements, such as yawning, closing eyes tightly and whilst clenching teeth (see Fig. 5).

Some participants used these movements to learn how to rumble in isolation (Fig. 5). However, most participants did not need to learn. Descriptions of how they realised they could rumble included *’I can’t explain it, [I] just once noticed I could do it. For me, it’s like flexing any other muscle in my body. I can’t imagine explaining this to someone, because to me it seems completely natural, just like waving a finger.’, ’I was trying to wriggle my ears and all I could hear was a rumbling’*.

#### Qualitative results

Both the majority of the neurological population (59%) and the general population (61%) stated an interest in controlling devices based on the EarSwitch concept. Among those who could rumble from the neurological population, 63% stated an in interest, however in the following question some participants stated they would not want to use it because they do no need AT.

**Fig. 6:**
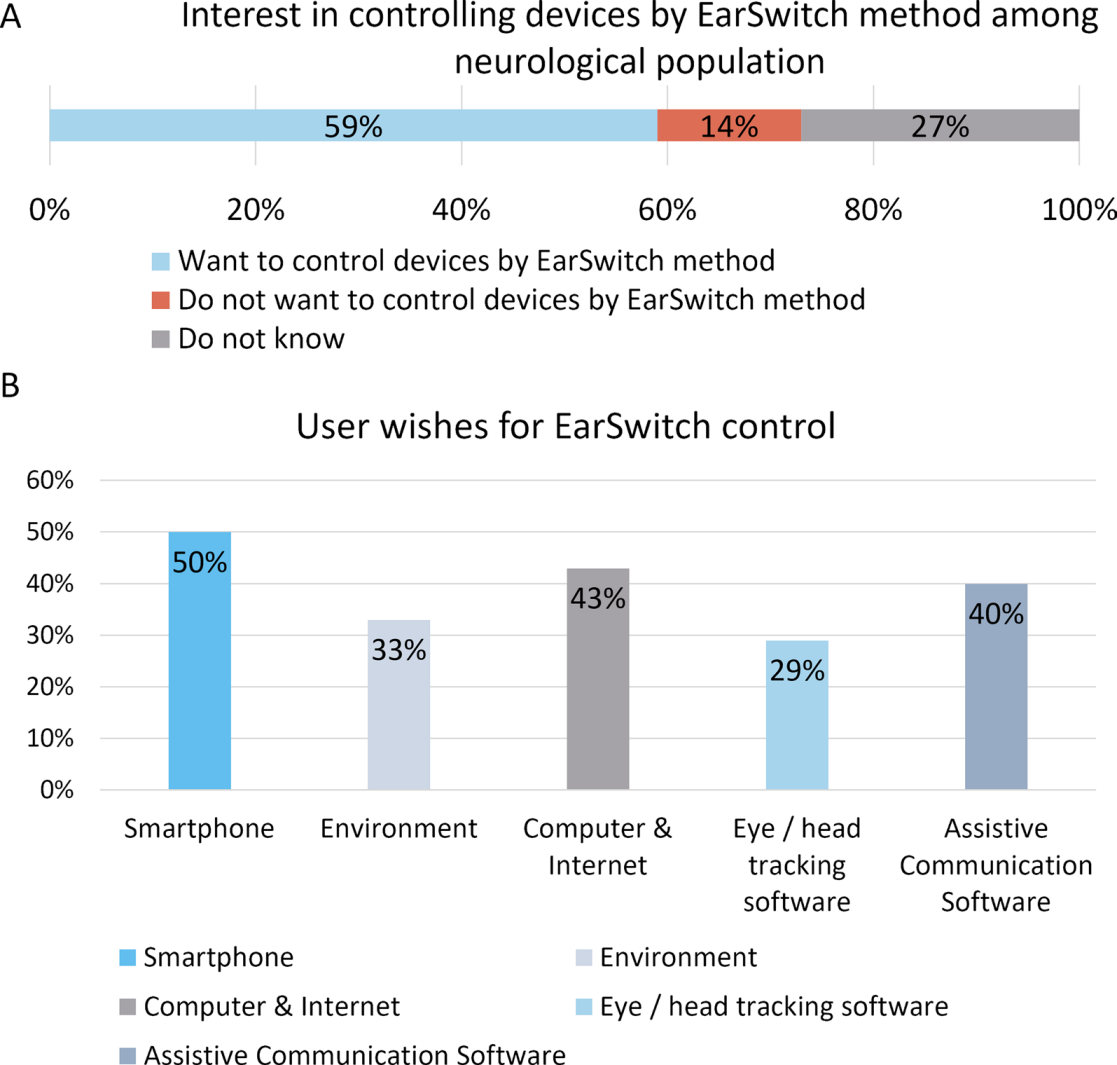
**A** Interest in controlling devices with technology based on the EarSwitch concept among the neurological population. **B** Top six things participants among neurological population named they would wish to control using rumbling

When asked what they wished to control from an earphone or hearing aid by rumbling, all possible options received votes (multi-choice question). The neurological population survey participants showed the highest interest in the possibility of controlling smartphones, followed by others (See Fig. 6). Among participants from the general population, the main function they wanted to use was to control earphones (63%), followed by smartphone functions (43%).

### Results of validation by recording visible eardrum movement by otoscope

Results of the Test 1 of the validation process are shown in Table 4 and 5. In the validation, 80% of the validation testing participants whom were self-reported ear rumblers exhibited visible eardrum movement. Eight participants were new rumblers (had not been aware of rumbling sensation before the survey, seven from the general population, and one from the population with neurological disorders). The validation found that four showed eardrum movement in isolation, including one who reported only being able to rumble with an accompanying trigger movement.

For the reliability of scoring eardrum movement among raters, the intraclass correlation coefficient demonstrated a high degree of reliability (average measure 0.94, $p<0.01$). The binomial test indicated that there was not a statistically significant difference (p = 0.376) between the researchers assessment during the test (0.8) and a majority view assessment from independent raters (0.9). (The results of the 3 independent raters for movement analysis can be found in Additional file 6).

**Table 4:** During the validation process, the data of those participants who did not have a clear view of their eardrum in either ear was deleted. This was mainly caused by earwax

	General population	Neurological population	All
Number of participants	88	4	92
No view of either eardrums (data removed)	12	1	13
Poor view of both eardrums (data removed)	8	1	9

**Table 5:** Results of validation. Validation was defined as visible movement of the eardrum while rumbling in isolation. Results are presented as the number of participants showing visible movement out of the total number of participants in each category

	General population	Neurological population	All
Participants with clear view	68	2	70
Validated movement in isolation: No. validated/ No. all participants from category (%)			
**Aware of rumbling pre-survey**	**51/61 (84%)**	**1/1 (100%)**	**52/62 (84%)**
Previously aware isolated rumbler	50/60 (83%)	1/1 (100%)	51/61 (84%)
Previously aware non-isolated rumbler	1/1 (100%)	0	1/1 (100%)
**Not aware of rumbling before survey**	**4/7 (57%)**	**0/1**	**4/8 (50%)**
New isolated rumbler	3/6 (50%)	0	3/6 (50%)
New non-isolated rumbler	1/1 (100%)	0/1	1/2 (50%)
**Total visible movement in isolation**	**55/68 (81%)**	**1/2 (50%)**	**56/70 (80%)**

#### Results of Test 2- Other actions causing visible movement of the eardrum

Each looking up, down, and to the left caused definite visible movement in one participant‘s eardrum and in two participant‘s eardrum by looking to the right and by eyebrow raising. However, in 16% (average across all actions) of the recordings, the assessor could not decide whether the eardrum moved.

#### Results of Test 3- Using EarSwitch concept as a binary switch control

The results of Test 3 can be seen in Table 6. Participants in the general population rated the target practice as either very easy (7/10) or easy (3/10). The same task was performed by one individual from the neurological population with Multiple Sclerosis, reporting the target practice as very easy. (The individual results can be found in Additional file 5.)

**Table 6:** Results of using the EarSwitch concept as a binary switch, the results were obtained in eight rounds (Test 3 of the Validation)

	No. participants	Mean response time	Mean No. extra hits
Participants from general population	9	1.57(±1.4) sec.	0.20 (±0.24)
Participant with Multiple Sclerosis	1	0.56 sec.	1.38

## Discussion

The findings suggest that self reported isolated rumbling is present among individuals with neurological disorders and self-reporting correlated well with objective rumbling in the general population. Results suggest EarSwitch is a promising alternative solution as an input for assistive technology.

### Ear rumbling differences between general and neurological populations

The significant difference observed in the proportion of isolated rumblers among the general and neurological populations (see Results of surveys) could be attributed to two potential explanations. First, a recruitment bias might have influenced the results. Second, individuals with neurological disorders may lose the ability to produce isolated rumbles.

Due to the recruitment method employed, which included participants who were aware that the survey was related to rumbling prior to participation, all the results should be regarded as upper bound prevelence. However, the proportion of participants who were familiar with the survey topic likely differs between the general and neurological populations.

It is assumed that most participants in the neurological population were recruited through organisations, where the recruitment material did not provide information about the primary aim of the study, while it is unknown how many participants from the general population were recruited who may have been biased to participate given the knowledge of what the survey was about (see Methods), i.e. increasing the likelihood of those who were aware of or able to ear rumble participating.

While this possibly explains the reason as to why the prevalence of the upper limit is 15% lower among the neurological population (Table 2), it does not explain the differences between the proportions of those who can rumble in isolation between the general and neurological population.

As discussed in Results, participants who discovered their ability to rumble during the survey were less likely to rumble in isolation. In the current study, those who were initially unaware of their ability to rumble were encouraged to make several attempts to produce the rumbling sensation. In contrast, the study by Roddiger et al. [55] did not provide participants with any information about the study’s focus during recruitment, and participants who could not rumble initially were not prompted to try deliberately during the survey. This difference in approach likely explains the difference in results between the two studies: in Roddiger et al.’s study, 80% of rumblers were able to rumble in isolation, a proportion more closely matching the isolation rate in the current study among participants who were already aware of their rumbling (69% in general population). However fewer new rumblers being able to rumble in isolation, cannot explain the difference between the two populations in the current study. As the ratio of new isolated rumblers is even lower in the neurological population (25%) compared to the general population (40%) (see Results).

The second explanation for the difference between the neurological population and the general population is that affected individuals lose the ability to rumble in isolation.

Despite the low number of participants with individual neurological disorders, at least one participant from each disorder with more than three participants reported being able rumble with some trigger movement, suggesting that some control of the muscle is present. However, there was no direct question asking if anyone lost their ability of rumbling.

Most participants from the neurological population (56%) had some form of MND, resulting in overall 95 participants with MND. 20% of participants with MND could rumble in isolation, indicating that rumbling maybe well preserved in some patients with MND (Table 3). Despite the high number of participants with MND, it is still difficult to identify whether all types of MND are resilient to the same extent, since most subjects did not specify the type they had (Fig. 4).

### EarSwitch as an input for assistive technology

EarSwitch provides invisible control for users and can be easily combined with other input controls because the ear is not commonly used for AT devices. The survey participants expressed interest in combining rumbling with other inputs to control AT, such as eye gaze, head-tracking or integration with existing user interface for patients with MND, such as Grid3 (Smartbox Assistive Technology, UK). Although, the greatest limitation of the EarSwitch concept as an AT input control is that it is only an available option for those who can rumble. However, non-isolated ear rumbling may be a satisfactory AT control method in patients with severe disabilities for whom any communication control may be acceptable. The finding that 70% report any form of rumbling (isolated or non-isolated) is encouraging. Our results indicate (see Table 2) that 30% of people are unaware of their ability to rumble. Interestingly, the ability to rumble was validated with a much lower rate for those people who were not aware of the phenomenom before the survey (50% validation rate of new rumblers opposed to 84% validation rate of rumblers who were aware of rumbling before the survey). This raises the question as to whether rumbling can be learnt. While this study did not focus on the trainability of ear rumbling, 27% and 31% of rumblers of the general population and neurological population claimed they learned to rumble in isolation by first rumbling with other trigger movements, for example yawning. This could suggest that first trying to increase awareness of the muscle with other accompanying movements could help train controlling the TTM (rumble), and could potentially lead to the ability to contract the muscle in isolation. In addition, small muscles are known to be much harder to control consciously, such as pelvic floor muscles, and special exercises have been developed to help people gain awareness of, and learn to control [62].

In terms of potential adverse consequences, a key consideration is the common symptom of fatigue in MND [63, 64]. It can significantly impair the sustained, controlled actions required for effective control of AT. However, this study and others to date have not investigated how tiring rumbling is or how its accuracy changes over time.

There is no reported evidence of long-term adverse consequences of repeated voluntary TTM contraction. There have been reports that Tonic Tensor Tympani Syndrome (TTTS) may be linked to a variety of symptoms including tinnitus, otalgia and hyperacusis, and resultant hypotheses suggesting it may mediate the symptoms of acoustic shock syndrome [65, 66]. In TTTS the TTM spontaneously rhythmically contracts and relaxes. In addition it has been suggested that the TTM contraction threshold decreases if the person is stressed, possibly increasing the probability of TTTS [66, 67]. Overall, there is no current evidence to suggest that frequent voluntary rumbling to control AT should cause adverse effects.

#### Sensing modalities

Reliable and robust sensing are important when developing AT input controls. In this study, an otoscope was used to sense rumbling in the ear and demonstrate proof of concept of using EarSwitch as a control input. However, the validation procedure highlighted a potential issue of camera-based approaches because 22 participants were excluded due to poor view of the eardrum (Table 4). This is because using a camera requires line of sight and the image must be positioned precisely on the ear drum with no occlusion (e.g. ear wax) while being robust to movement (e.g. of the head or jaw). Röddiger et al. used a pressure sensor in a sealed ear canal to detect movement, but a long-term sealed ear canal could lead to complications (e.g. ingress of microorganisms) and would block sound which could isolate the user more [55]. In the future, other modalities may provide better alternatives to detect ear rumbling that address the line of sight issue, while making sure the user has unimpaired hearing.

Previous research has not exhaustively investigated which other movements could accidentally trigger a rumble; however, this is important from a detection perspective. Participants were asked to name all movements that made them feel a rumbling sensation. Notably, the general population and those with neurological disorders followed a similar trend regarding the accompanying movements that made them feel a sensation of rumbling. In both groups, the majority of participants rumbled while yawning, followed by closing their eyes tightly, opening their mouths wide, and clenching their teeth (Figure 6).

### Strengths and limitations of the study

The strength of this study is the large number of participants recruited from both the general population (n=1853) and patients with neurological disorders (n=170). However, the recruitment process, where some participants may have been drawn from a subreddit dedicated to ear rumbling, may have introduced bias, potentially inflating prevalence rates among those able to rumble who were already aware of this ability.

The main limitation of this study is that we only had a small number of participants to validate the concept in the neurological population; however, findings in the general population show that self-reporting correlates well with objective rumbling, and it seems likely that this can be translated to the neurological population. It is believed the ability to correctly identify if someone can rumble does not depend on the physical condition, but this needs to be validated with larger studies.

## Conclusion

This is the first study investigating whether the ability to rumble is preserved among the patients with neurological disorders and the possibility of using it for controlling assistive technology. The results suggest that self-reported rumbling is present in patients with neurological disorders, particularly those affected by MND. During this study participants from the general population also showed that after reading a description of ear rumbling, without experiencing any feedback, participants were able to identify with a high accuracy (81%) their ability to rumble. It is predicted that this validation rate is likely to be transferable to the neurological population as well. However, further studies are needed to determine how well control of the muscle is preserved in the last stages of the disease, or to identify the underlying biological mechanisms behind resilience in MND.

Rumbling was successfully sensed by picking up the movement of the eardrum using an otoscope placed in the ear canal. The viability of the EarSwitch concept; using rumbling sensed by an otoscope to control a computer interface was proven in a study with ten participants, despite using a motion detection threshold that was not personalised for each user. Improved results would be anticipated with the further development of the sensing method and customised sensitivity calibrations.

Apart from results suggesting control of the muscle is present and movement being easily picked up with a small device, participants also expressed their interest in controlling devices by the EarSwitch method. Overall, the results of this study demonstrate that a significant proportion of the population with neurological disorders could benefit from assistive technology controlled by using a new method based on the EarSwitch concept.

## Supplementary Information

Supplementary file 1.

Supplementary file 2.

Supplementary file 3.

Supplementary file 4.

Supplementary file 5.

Supplementary file 6.

## Data Availability

All anonymised data collected from both surveys can be found in Additional files 3 and 4. As well as the detailed anonymised results of the Validation.

## Appendix

See Tables 7, 8

**Table 7:** Demographics of participants taking part in surveys (Phase 1 and Phase 2)

	General population n(%)	Neurological population n(%)
Total	1853 (100%)	170 (100%)
Age group	Gender distibution	
18-24	222 (12%)	10 (6%)
25-34	296 (16%)	22 (13%)
35-44	352 (19%)	26 (15%)
45-54	204 (11%)	14 (8%)
55-64	333 (18%)	32 (19%)
65-74	296 (16%)	37 (22%)
Over 75	37 (2%)	3 (2%)

Gender	Gender Distribution	
Female	982 (53%)	73 (43%)
Male	852 (46%)	94 (55%)
Other	19 (1%)	3 (2%)

Age group	Age onset of rumbling	
Under 6	204 (11%)	32 (19%)
6-11	407 (22%)	49 (29%)
12-17	296 (16%)	24 (14%)
18-24	593 (32%)	29 (17%)
25-34	37 (2%)	8 (5%)
35-44	19 (1%)	2 (1%)
45-54	37 (2%)	5 (3%)
55-64	56 (3%)	14 (8%)
65-74	74 (4%)	3 (2%)
Couldn’t remember	130 (7%)	3 (2%)

**Table 8:** Demographics of participants taking part in validation (Phase 3)

Age group	Age distribution n(%) (N=70)
18-24	1 (1%)
25-34	5 (7%)
35-44	4 (6%)
45-54	18 (26%)
55-64	22 (31%)
65-74	17 (24%)
Over 75	3 (4%)

Gender	Gender Distribution n(%)
Male	34 (49%)
Female	36 (51%)

## Abbreviations

AAC: Augmentative and alternative communication
ALS: Amyotrophic lateral sclerosis
AT: Assistive technology
BCI: Brain-computer interface
EEG: Electroencephalography
EMG: Electromyography
EOG: Electrooculogram
sec: Seconds
MND: Motor Neurone disease
MS: Multiple sclerosis
PBP: Progressive bulbar palsy
PLS: Primary lateral sclerosis
REM: Rapid eye movement
TTM: Tensor tympani muscle
TTTS: Tonic tensor tympani syndrome
