# Supplementary material for: Exploring the ’EarSwitch’ concept: a novel ear based control method for assistive technology
Source: J Neuroeng Rehabil. 2024 Dec 2;21:210. doi: 10.1186/s12984-024-01500-z (PMC11613744; doi:10.1186/s12984-024-01500-z)
Supplement: Supplementary file 1 [file 12984_2024_1500_MOESM1_ESM.pdf]

Earswitch for the general population

Introduction (part 1)

The purpose of this survey is to establish what proportion of the general population are able to move a particular muscle in their ear; something that you and many others may never have tried to do before.

This is important to know because there is now some early evidence that this muscle can be used to control electronic communication tools such as an assistive keyboard (like the one Stephen Hawking used). For people with conditions affecting their speech, communicating with friends and loved ones can be extremely difficult and sometimes impossible. This technology, which is being researched at the University of Bath is hoping to aid a range of people with severe communication difficulties.

The survey should take around 5 minutes to complete.

Participant information

This section forms part of the process of informed consent. It should give you the basic idea of what the research is about and what your participation will involve. Please read the following information carefully:

- **What is the purpose of this research project?** The purpose of this survey is to try and find out what proportion of the general population are able to make changes within their ears by moving a particular muscle in the ear; something that you and many others may never have tried to do before. This research is being conducted with the aim of developing better technological tools to help people with communication problems (such as those with motor neurone disease).
- **Who can be a participant?** Anyone aged 18 or over can take part.
- **Do I have to take part?** It is completely up to you to decide if you would like to participate.
- **What are the exclusion criteria?** Individuals under the age of 18 are unable to take part in this particular study.
- **What are the possible benefits of taking part?** There will be no immediate direct benefits of you taking part, However, the information that you and other participants provide will help us to understand what proportion of the general population can voluntarily contract this muscle in your ear and may help to develop technology that would assist individuals with severe neurological disorders (such as motor neurone disease) to communicate more easily in the future.
- **What are the possible disadvantages and risks of taking part?** There are no disadvantages to you taking part in this project. Should you not want to answer a question, you can choose not to answer and exit the survey at any time.
- **What will happen to my data?** Only the University of Bath researchers will have access to this data and will be treated as confidential at all times. Any personal or identifiable data will be kept in a locked room or on a password-protected file on a university of Bath's secure server. The storage of data will be undertaken in accordance with GDPR. It is possible that the results of this survey will be published in peer-reviewed scientific journals and/or presented at academic conferences.
- **Can I change my mind about participating?** Yes, you can change your mind about participating at any time. Whilst completing the survey, you can stop by simply closing the web browser. If you have already completed the survey, you are free to withdraw your consent within 2 weeks without having to give a reason. Please send an email (from the email address you provided in the survey) to either Dr. D. Cazzola (dc547@bath.ac.uk) or Dr. R. Stevenson (rdms20@bath.ac.uk) requesting your data be removed.
- **What should I do if I require further information?** You can contact the project

supervisor Dr. Dario Cazzola (dc547@bath.ac.uk) or the Chair of the University's ethics committee for health research, Professor James Betts (j.betts@bath.ac.uk)

Consent

1. Are you willing to continue and participate in this survey? ✱ *Required*

- ○ Yes
- ○ No

Thank you

Thank you for showing interest in this research project

Introduction (part 2)

We know that some people can make a special sound or sensation in their ears either intentionally or when they perform certain movements, such as yawning, closing their eyes tightly, clenching their teeth, or opening their mouth wide. **Please note, this IS NOT the same sensation as 'equalising' the pressure in your ears (when descending on an aeroplane for example). Nor is it hearing a brief “click” (called a Eustachian tube click)**. It's a "rumbling" or "fluttering" noise / sensation, like the sound of distant thunder, or a muffling / dampening of your hearing.

Whether or not you've ever experienced this sensation, we'd like to ask you some questions about it. Throughout this survey, we will refer to these changes as **"rumbling"**.

If you like, you can watch the short 20-second video which explains this in a little more detail (or just skip to the next section):

*modified from ScienceABC

Awareness (part 1)

2. Now that you've read the description above, have you **previously** been aware of "rumbling" as something you experience? ✱ *Required*

- ○ Yes
- ○ No

Awareness (part 2)

3. At approximately what age were you when you became aware of the "rumbling" / fluttering/ muffling sensations or noise? ✱ *Required*

- ◯ Under 6 years
- ◯ 6 to 11 years
- ◯ 12 to 17 years
- ◯ 18 to 24 years
- ◯ 25 to 34 years
- ◯ 35 to 44 years
- ◯ 45 to 54 years
- ◯ 55 to 64 years
- ◯ 65 to 74 years
- ◯ 75 years or over
- ◯ Can't remember

4. In what situations did you notice the "rumbling"/ fluttering/ muffling sensations or noise? (Check all that apply) ✱ *Required*

- ☐ While yawning
- ☐ While closing eyes tightly
- ☐ While clenching teeth
- ☐ While opening mouth wide
- ☐ In isolation (i.e. I can do it without any other movements)
- ☐ Other

4.a. If you selected Other, please specify:

5. If you already knew you could “rumble” in isolation (i.e. without any other movements/ such as yawning etc) please describe how you learned to “rumble” in isolation (please tick all appropriate answers): *Optional*

- [ ] I can “rumble” in isolation (I.e. without other movements), and found that I could just do it without training
- [ ] I noticed that I could “rumble” at the same time as other movements, such as yawning or eye closing etc, and then learnt how to do it in isolation (I.e. without other movements)
- [ ] I learnt how to rumble in isolation (I.e. without other movements) from someone else (either personally or on the internet).

6. If you learned how to “rumble” in isolation (i.e. without any other movement/ yawning etc), please describe below how you learned this, as if you were training another person to do it? *Optional*

Trying it

7. Please try the following movements one at a time (Yawning, Closing your eyelids tightly for a few seconds, Clenching your teeth for a few seconds, Opening your mouth wide). Concentrate on any effect that you notice in your ears. **We are interested in “rumbling”, and not an “equalisation” effect, nor hearing a brief “click" sound.** Did you feel the "**rumbling**" sensation with any of these actions? ✱ *Required*

- ○ Yes
- ○ No

7.a. If you did, which action (or actions) brought on the rumbling sensation? (please tick all that apply).

- ☐ Whilst yawning
- ☐ Whilst closing my eyelids tightly for a few seconds
- ☐ Whilst clenching my teeth for a few seconds
- ☐ Whilst opening my mouth wide

8. Please would you try making the "rumbling" sensation without doing any of the movements previously tried? Can you still do it? ✱ *Required*

- ○ Yes
- ○ No

Applications of ear rumbling (part 1)

9. Would you be interested in controlling your earphones, smartphone, hearing aid or computer by ear “rumbling“ (i.e. controlling a muscle in your ear) if you could learn to do it in isolation? ✱ *Required*

- Yes
- No
- Dont know

Applications of ear rumbling (part 2)

10. Which technologies or uses would you personally wish to control using an earphone or hearing-aid with voluntary ear control ("rumbling") (please select all that apply)? ✱ *Required*

- ☐ Earphones (e.g. volume control, change track)
- ☐ Smartphone functions
- ☐ Sports type helmet camera
- ☐ Control a computer as a switch to “select” with eye- or head-tracking
- ☐ Assistive technology software (e.g. for people with limited communication)
- ☐ Hearing aids (e.g. volume control)
- ☐ Cochlear implants (e.g. volume control)
- ☐ Gaming
- ☐ None of the above
- ☐ Don’t know

11. Is there any other function, device or application that you can suggest would benefit from being controlled by an ear “rumble” from an earphone or hearing-aid?

Further involvement

12. If you are in the UK and close to Bath, would you be interested in taking part in further research on this topic? Researchers at the University of Bath are interested to find out more about how and when this muscle moves in a small sample of those who have completed the survey. You would need to come to the University of Bath for around 1 hour to perform these movements again with a small camera in your ear. Don't worry this won't hurt and we will ensure you are fully protected during your visit. We will ask for you contact details later on if you are interested. ✱ *Required*

- ○ Yes
- ○ No

About you

13. 4. What is your age? ✱ *Required*

- Under 18
- 18 to 24
- 25 to 34
- 35 to 44
- 45 to 54
- 55 to 64
- 65 to 74
- 75 or over

14. What is your gender?

- Male
- Female
- Other

15. What is your occupation / main activity (optional)?

Contact details

16. (Optional) Please provide your email address in case you change your mind about participating (you will have 2 weeks to do this from submitting this survey). We will use your email address to identify you and your data. *Optional*

Completed

Thank you for taking part in this survey.

Need survey respondents? Click this link to receive credits that earn you free respondents at SurveySwap.io. --> https://surveyswap.io/sr/aVkfDFlx6TU6YrVe

---
