# Supplementary material for: Exploring the ’EarSwitch’ concept: a novel ear based control method for assistive technology
Source: J Neuroeng Rehabil. 2024 Dec 2;21:210. doi: 10.1186/s12984-024-01500-z (PMC11613744; doi:10.1186/s12984-024-01500-z)
Supplement: Supplementary file 2 [file 12984_2024_1500_MOESM2_ESM.pdf]

Earswitch for people with neurological conditions

Introduction (part 1)

The purpose of this survey is to try and find out what proportion of people **with neurological conditions** are able to make changes within their ears by moving a particular muscle in the ear; something that you and many others may never have tried to do before.

This research is being conducted by the University of Bath with the aim of developing better technological tools to help people with communication problems.

The survey should take no more than 5-10 minutes to complete.

Participant information

This section forms part of the process of informed consent. It should give you the basic idea of what the research is about and what your participation will involve. Please read the following information carefully:

- **What is the purpose of this research project?** The purpose of this survey is to try and find out what proportion of people are able to make changes within their ears by moving a particular muscle in the ear; something that you and many others may never have tried to do before. This research is being conducted with the aim of developing better technological tools to help people with communication problems.
- **Who can be a participant?** Anyone aged 18 years and over, who has a recognised neurological condition.
- **Do I have to take part?** It is completely up to you to decide if you would like to participate.
- **What are the exclusion criteria?** Individuals under the age of 18 are unable to take part in this particular study.
- **What are the possible benefits of taking part?** There will be no immediate direct benefits of you taking part, However, the information that you and other participants provide will help us to understand what proportion of a population with motor neurone disease can voluntarily contract this muscle in your ear and may help to develop technology that would assist individuals with severe neurological disorders to communicate more easily in the future.
- **What are the possible disadvantages and risks of taking part?** There are no disadvantages to you taking part in this project. Should you not want to answer a question, you can choose not to answer and exit the survey at any time.
- **What will happen to my data?** Only the University of Bath researchers will have access to this data and will be treated as confidential at all times. Any personal or identifiable data will be kept in a locked room or on a password-protected file on a university of Bath's secure server. The storage of data will be undertaken in accordance with GDPR. It is possible that the results of this survey will be published in peer-reviewed scientific journals and/or presented at academic conferences.
- **Can I change my mind about participating?** Yes, you can change your mind about participating at any time. Whilst completing the survey, you can stop by simply closing the web browser. If you have already completed the survey, you are free to withdraw your consent within 2 weeks without having to give a reason. Please send an email (from the email address you provided in the survey) to either Dr. D. Cazzola (dc547@bath.ac.uk) or Dr. R. Stevenson (rdms20@bath.ac.uk) requesting your data be removed.
- **What should I do if I require further information?** You can contact the project

supervisor Dr. Dario Cazzola (dc547@bath.ac.uk) or the Chair of the University's ethics committee for health research, Professor James Betts (j.betts@bath.ac.uk)

Consent

1. Are you willing to continue and participate in this survey? ✱ *Required*

- ○ Yes
- ○ No

Thank you

Thank you for showing interest in this research project

Introduction (part 2)

We know that some people can make a special sound or sensation in their ears either intentionally or when they perform certain movements, such as yawning, closing their eyes tightly, clenching their teeth, or opening their mouth wide. **Please note, this IS NOT the same sensation as 'equalising' the pressure in your ears (when descending on an aeroplane for example). Nor is it hearing a brief “click” (called a Eustachian tube click)**. It's a "rumbling" or "fluttering" noise / sensation, like the sound of distant thunder, or a muffling / dampening of your hearing.

Whether or not you've ever experienced this sensation, we'd like to ask you some questions about it. Throughout this survey, we will refer to these changes as **"rumbling"**.

Please watch the following video which explains this in a little more detail:

*modified from ScienceABC

Awareness (part 1)

2. Now that you've read the description above, have you **previously** been aware of "rumbling" as something you experience? ✱ *Required*

- Yes
- No

Awareness (part 2)

3. Approximately what age were you when you became aware of the "rumbling"/ fluttering/ muffling or dampening sensation or noise? ✱ *Required*

- Under 6 years
- 6 to 11 years
- 12 to 17 years
- 18 to 24 years
- 25 to 34 years
- 35 to 44 years
- 45 to 54 years
- 55 to 64 years
- 65 to 74 years
- 75 years or over
- can't remember

4. In what situations did you notice the "rumbling"/ fluttering/ muffling sensations or sounds? (Check all that apply) ✱ *Required*

- [ ] While yawning
- [ ] While closing eyes tightly
- [ ] While clenching teeth
- [ ] While opening mouth wide
- [ ] In isolation (i.e. I can do it without any other movements)
- [ ] Other

4.a. If you selected Other, please specify:

5. If you already knew you could “rumble” in isolation (i.e. without any other movements/ such as yawning etc) please describe how you learned to “rumble” in isolation (please select one answer): *Optional*

- ○ I can “rumble” in isolation (I.e. without other movements), and found that I could just do it without training
- ○ I noticed that I could “rumble” at the same time as other movements, such as yawning or eye closing etc, and then learnt how to do it in isolation (I.e. without other movements)
- ○ I learnt how to rumble in isolation (I.e. without other movements) from someone else (either personally or on the internet).

6. If you learned how to “rumble” in isolation (i.e. without any other movement/ yawning etc), please describe below how you learned this, as if you were training another person to do it? *Optional*

Trying it

7. **If you are able**, please try the following movements one at a time (Yawning, Closing your eyelids tightly for a few seconds, Clenching your teeth for a few seconds, Opening your mouth wide). Concentrate on any effect that you notice in your ears. **We are interested in "rumbling", and not an "equalisation" effect, nor hearing a brief "click" sound.** Did you feel the "**rumbling**" sensation with any of these actions? ✱ *Required*

- ○ Yes
- ○ No

7.a. If you did, which action (or actions) brought on the rumbling sensation? (please tick all that apply).

- ☐ Whilst yawning
- ☐ Whilst closing my eyelids tightly for a few seconds
- ☐ Whilst clenching my teeth for a few seconds
- ☐ Whilst opening my mouth wide

8. Please would you try making the "rumbling" sensation without doing any of the movements previously tried? Can you still do it? ✱ *Required*

- ○ Yes
- ○ No

Assistive use (part 1)

9. Do you currently use, or feel you would benefit from assistive technology (electronic devices for communication or controlling your mobility or environment)? ✱ *Required*

- ◯ Yes, I currently use assistive technology
- ◯ No, I don't currently use, but think I could benefit from assistive technology
- ◯ No, I don't currently use, and I don't think I'd benefit from assistive technology
- ◯ Unsure / don't know

Assistive use (part 2)

10. Which assistive technology devices or methods do you currently use (Please select all that apply)?

- ☐ Eye-tracking
- ☐ Button switch
- ☐ Sip & puff switch
- ☐ Joystick
- ☐ Sound switch
- ☐ Speech
- ☐ Other

10.a. If you selected Other, please specify:

11. If you currently use assistive technology, which activities do you use them for (Please select all that apply)? ✱ *Required*

- ☐ Communication
- ☐ Mobility
- ☐ Controlling your environment (e.g. light, TV etc)
- ☐ Controlling a computer
- ☐ Gaming
- ☐ Internet use
- ☐ Other

11.a. If you selected Other, please specify:

Applications of ear rumbling (part 1)

12. Would you be interested in controlling any device or technology, from an earphone or hearing-aid, by ear “rumbling”? ✱ *Required*

- ◯ Yes
- ◯ No
- ◯ Don't know

Applications of ear rumbling (part 2)

13. What device or function or application would you wish to control from an earphone or hearing aids by ear “rumbling” (please select all that apply)?: ✱ *Required*

- [ ] Assistive communication software
- [ ] “Select” control for eye-tracking or head-tracking
- [ ] Mobility e.g. wheelchair
- [ ] Control your environment
- [ ] Control a computer
- [ ] Earphones (e.g. volume, change track)
- [ ] Smartphone functions
- [ ] Hearing aids (e.g. volume control)
- [ ] Cochlear implants (e.g. volume control)
- [ ] Gaming
- [ ] Internet use
- [ ] All of the above
- [ ] None of the above

Other functions?

14. Is there any other function, device or application that you can suggest would benefit from being controlled by an ear “rumble” from an earphone or hearing-aid?

About you

15. What is your age? ✱ *Required*

- Under 18
- 18 to 24
- 25 to 34
- 35 to 44
- 45 to 54
- 55 to 64
- 65 to 74
- 75 or over

16. What is your gender?

- Male
- Female
- Other

17. If you have a neurological condition, please would you describe your condition (if you are willing to share this).

18. Please would you describe your current ability/ activity or occupation (if you are willing to share this)?

Contact details

19. If you are in the UK and close to Bath, would you be interested in taking part in further research on this topic? Researchers at the University of Bath are interested to find out more about how and when this muscle moves in a small sample of those who have completed the survey. You would need to come to the University of Bath for around 1 hour to perform these movements again with a small camera in your ear. Don't worry this won't hurt and we will ensure you are fully protected during your visit. We will ask you to provide your contact details in the next question if you are interested. ✱ *Required*

- ◯ Yes
- ◯ No

20. Please provide your email address if you have indicated that you would like to be involved in further reserarch or in case you change your mind about participating (you will have 2 weeks to do this from submitting this survey). In this case we will use your email address to identify you and your data. *Optional*

Completed

Thank you for completing the survey
