# Supplementary material for: Exploring the ’EarSwitch’ concept: a novel ear based control method for assistive technology
Source: J Neuroeng Rehabil. 2024 Dec 2;21:210. doi: 10.1186/s12984-024-01500-z (PMC11613744; doi:10.1186/s12984-024-01500-z)
Supplement: Supplementary file 6 [file 12984_2024_1500_MOESM6_ESM.pdf]

Independent assessor scores from the independent movement analysis:

**Video**	**Assessor 1**	**Assessor 2**	**Assessor 3**	**Movement Y/N (Majority)**	**Movement binary score**
1	2	3	2	Y	1
2	1	1	1	Y	1
3	2	2	1	Y	1
4	1	1	1	Y	1
5	2	3	2	Y	1
6	3	3	3	Y	1
7	0	0	0	N	0
8	3	3	3	Y	1
9	2	3	2	Y	1
10	1	2	2	Y	1
